# Geographic and Environmental Differences in Associations Between Food Insecurity and Nutritional and Inflammatory Biomarkers Among Pregnant Participants in the *All of Us* Research Program

**DOI:** 10.3390/nu18182965

**Published:** 2026-09-10

**Authors:** Alaijah Bashi, Viviane Schuch, Amber Dawning, Tyana T. Joseph, Peter Baltrus, Carla J. Moore, Erica L. Johnson

**Affiliations:** 1 Microbiology, Biochemistry and Immunology, Morehouse School of Medicine, Atlanta, GA 30310, USAerijohnson@msm.edu (E.L.J.); 2 Community Health and Preventative Medicine, Morehouse School of Medicine, Atlanta, GA 30310, USA; 3 Clinical Research Center, Morehouse School of Medicine, Atlanta, GA 30310, USA

**Keywords:** food insecurity, pregnancy outcomes, nutritional status, maternal health, food environment, geographic health disparities

## Abstract

Background/Objectives: Food insecurity (FI) affects a substantial proportion of pregnant individuals in the United States and varies across geographic contexts. Rural and urban communities differ in food access, socioeconomic conditions, and environmental exposures, yet how these differences relate to maternal biology remain misunderstood. Methods: This study examined associations between FI, geographic context, and metabolic, nutritional, and inflammatory biomarkers among pregnant participants in the Southeastern United States (SE-US) using data from the *All of Us* (*AoU*) Research Program. Survey, clinical, and laboratory data were integrated with ZIP3-level geographic and food environment indicators. Analyses were conducted using biomarker-specific complete-case cohorts and multivariable regression models. Results: This study found that FI was more prevalent among rural participants than urban participants. However, in the overall cohort, FI was associated with higher body mass index, higher non-fasting glucose, higher C-reactive protein, and lower high-density lipoprotein cholesterol. Pregnant food-insecure participants also exhibited lower concentrations of vitamin B12, albumin, vitamin D, and folate, while ferritin did not differ. Stratified analyses demonstrated variability in these associations by urbanicity, with stronger and more consistent differences observed in urban populations and fewer significant differences in rural settings. Conclusions: Limitations of this study include potential residual confounding, EHR-related selection bias, exposure misclassification, and limited generalizability. Despite the limitations, recent self-reported household FI, assessed at enrollment and referring to the preceding 12 months, was associated with differences in several metabolic and nutritional biomarkers, with patterns varying by geographic context. Further longitudinal research is needed to clarify these associations and inform targeted interventions.

## 1. Introduction

Food insecurity (FI), defined as limited or uncertain access to nutritionally adequate, safe, or culturally appropriate foods [1], remains a pervasive public health concern in the United States [2]. In 2023, 13.5% of U.S. households (approximately 18.0 million households) experienced FI, representing an increase from 12.8% in 2022 [2]. Pregnant individuals face an elevated risk of FI due to increased physiological demands, economic strain, and shifts in household food dynamics during pregnancy [3,4]. National surveillance systems consistently estimate that approximately 1 in 5 pregnant individuals may experience FI, with an even higher prevalence among Black, Hispanic/Latinx, and low-income communities. This includes estimates of FI affecting 23.3% of Black households and 21.9% of Hispanic households, compared with the national average of 13.5% [5,6]. These disparities reflect broader patterns of social and economic variation and have critical implications for maternal health [5]. Recent self-reported household FI has been linked to a range of adverse outcomes, including hypertensive disorders of pregnancy, excessive gestational weight gain, preterm birth, and low birthweight [6]. Together, these findings acknowledge FI as both a socioeconomic challenge and a clinically relevant risk factor during gestation [5,6].

Although the associations between FI and adverse pregnancy outcomes are well-documented, the metabolic pathways underlying these relationships remain insufficiently understood [7,8]. Some studies in pregnant and non-pregnant populations have shown that limited nutritional intake, constrained diet quality, and chronic stress, which are key features of FI, may influence micronutrient status, inflammatory responses, and metabolic regulation [9,10]. Prior research outside of pregnancy has shown that FI is associated with deficiencies in iron, Vitamin B12, Vitamin D, and folate, as well as elevated markers of systemic inflammation such as C-reactive protein (CRP) [11,12,13]. However, evidence among pregnant populations is sparse, fragmented, and often limited by small sample sizes or lack of biomarker data [3]. As a result, the extent to which FI is associated with measurable disruptions in maternal nutritional or inflammatory biomarkers during pregnancy is not well-characterized [14].

FI does not occur in isolation; it is shaped by the structural and neighborhood contexts in which individuals live [15]. Access to healthy foods, exposure to psychosocial stressors, and availability of social and material resources vary widely across communities and collectively define the neighborhood’s socioeconomic environment [15,16]. In the U.S. census-designated Southeastern region of the United States (SE-US), the relationship between individual FI and the local food environment, characterized by “food swamps,” high poverty, and transportation barriers, creates a complex landscape of risk, especially during pregnancy. Rural areas often face severe resource deprivation and reduced access to healthy food infrastructure, whereas urban environments may offer greater access to fortified but highly processed foods [17], potentially buffering certain nutritional deficiencies while exacerbating metabolic stress [16,18]. Despite evidence linking recent self-reported household FI to adverse maternal and fetal outcomes, relatively few studies have employed multilevel frameworks that integrate individual-level exposure with contextual factors, such as urbanicity, neighborhood deprivation, transportation access, and environmental stressors, to examine their combined influence on maternal inflammatory and metabolic responses during pregnancy [6,8,19,20,21,22]. There is a need for multilevel investigations that integrate individual-level FI with neighborhood-level indicators of socioeconomic disadvantage to understand how “place” is associated with biological vulnerabilities. These vulnerabilities encompass differences in biomarker profiles that may indicate poorer nutritional status, metabolic health, or inflammatory burden among food-insecure participants during gestation.

To address these critical gaps, this study leverages the *All of Us* (*AoU*) Research Program, a large and diverse national cohort, to investigate associations between recent self-reported household FI, assessed at enrollment and referring to the preceding 12 months, and nutritional and inflammatory biomarkers measured during pregnancy. Specifically, this study examined ferritin, Vitamin B12, Vitamin D, folate, albumin, and CRP, markers that reflect iron status, micronutrient reserves, protein status, and systemic inflammation, focusing specifically on maternal health during pregnancy [23,24]. Additionally, this study incorporated ZIP3-code level indices, including Rural-Urban Commuting Area (RUCA) urbanicity codes and the Grocery Gap Atlas, to examine how geographic context and local food environments may be associated with differences in biological vulnerability. This research provides critical insight into the structural conditions that shape maternal cardiometabolic health and highlights the need for location-specific interventions to translate discovery into clinical and public health applications.

This study provides a framework for exploring associations between geographic context and maternal nutritional, metabolic, and inflammatory biomarker profiles during pregnancy. By identifying the distinct effects of rural and urban food environments on maternal biomarkers, this study suggests that these factors may be associated with less favorable maternal nutritional and inflammatory biomarker profiles during pregnancy [25]. Ultimately, this research may help bridge the gap between social determinants of health and precision medicine by supporting a dual-focus strategy that considers both the patient’s clinical status and the environmental context that influences their health.

## 2. Materials and Methods

### 2.1. Data Integration and Study Population

The study population was derived from the *AoU* Research Program (CDR v7; C2022Q4R13), a nationwide initiative designed to advance precision medicine by enrolling participants from populations historically underrepresented in biomedical research [26]. *AoU* provides a multimodal data architecture, integrating self-reported survey responses, physical measurements, and electronic health records (EHRs) with biospecimen-derived laboratory data [26]. By leveraging this dataset, this cross-sectional analysis bridges individual-level clinical biomarkers, including micronutrient reserves, protein status, and systemic inflammation, with broader social determinants of health (SDOH).

This cross-sectional study utilized baseline data from pregnant participants enrolled in the *AoU* Research Program between May 2018 and 1 July 2022. Participants were identified using the Pregnant Participants cohort definition available within the *AoU* Researcher Workbench Cohort Builder. Pregnancy status was not determined using a study-specific algorithm; rather, participants were selected from the standardized *AoU* pregnancy cohort generated using participant survey and EHR data.

The Southeastern United States (SE-US) was selected because it experiences a disproportionately high burden of food insecurity (FI) and socioeconomic disadvantage relative to many other regions of the United States [27]. Eligible participants were required to (1) belong to the *AoU* Pregnant Participants cohort, (2) reside within the predefined SE-US study region based on available geographic data, (3) complete the validated two-item Hunger Vital Sign FI assessment, and (4) have at least one eligible EHR-derived biomarker measurement.

The initial national cohort identified through the Researcher Workbench consisted of 85,149 pregnant participants who answered both questions in the validated FI questionnaire and had at least one eligible biomarker measurement available. Geographic restriction to the predefined SE-US region yielded a final analytic cohort of 12,192 participants. Application of the prespecified geographic restriction to the Southeastern United States excluded 72,957 otherwise eligible participants, resulting in a final analytic cohort of 12,192 participants. Biomarker-specific complete-case analyses were subsequently conducted because laboratory availability varied across outcomes. The final sample substantially exceeded the estimated sample size required to detect small-to-moderate associations in both continuous and binary outcomes, providing adequate statistical power for the planned analyses (80% power and α = 0.05).

Biomarker analyses were conducted using biomarker-specific complete-case datasets, resulting in varying sample sizes across outcomes due to differences in laboratory data availability. Participants missing FI or geographic classification data were excluded from the analytic cohort. All biomarker and clinical measurements were obtained at or near enrollment in the *AoU* Research Program. The final analytic sample consisted of pregnant participants who met all study eligibility criteria (Table 1).

Because this study was restricted to participants residing in the SE-US, the final cohort reflects a study-design geographic restriction rather than selective participant exclusion. Nevertheless, potential selection bias related to voluntary participation in the *AoU* Research Program and the availability of EHR-derived laboratory measurements cannot be excluded. As a secondary analysis of an existing cohort, this study was also limited by the variables available within the *AoU* dataset, which was not specifically designed to investigate biomarker profiles associated with FI during pregnancy. Consequently, several potentially important pregnancy-related clinical and behavioral factors were unavailable or incompletely captured. Table 1 summarizes the demographic, socioeconomic, and geographic characteristics of the analytic cohort and provides context for interpreting differences between food-secure and food-insecure participants in subsequent adjusted analyses.

### 2.2. Food Insecurity Assessment

FI was assessed in pregnant participants at enrollment using a validated, standardized two-question screening instrument derived from the U.S. Department of Agriculture (USDA) food security assessment [28]. Participants were classified as food-insecure if they responded affirmatively to either of the following statements: (1) “*Within the past 12 months, we worried whether our food would run out before we got money to buy more.*” Or (2) “*Within the past 12 months, the food we bought just didn’t last and we didn’t have money to get more.*” These questions are consistent with established screening practices [28]. FI was analyzed as a binary exposure (food-insecure vs. food-secure) in all primary analyses. FI status was derived from participant self-reported responses to the Hunger Vital Sign questionnaire administered through the *AoU* survey platform. FI was not obtained from external datasets and was not independently verified; therefore, findings should be interpreted in the context of participant-reported household FI during the preceding 12 months. Additionally, the measure may reflect FI occurring before pregnancy, during pregnancy, or both, depending on the timing of enrollment. Because the FI assessment and biomarker measurements were obtained at or near enrollment, FI was treated as a contemporaneous indicator of recent household FI in relation to measured biomarker outcomes.

### 2.3. Geospatial and Socioeconomic Characterization

To characterize the broader environmental context, participant data were linked to external datasets capturing food access and economic conditions within the *AoU* Secure Cloud Computing Platform using ZIP3-level geographic identifiers. In addition, Geographic Information Systems (GIS) mapping was employed using R-Studio (R-4.6.0) to visualize the distribution of FI across urban and rural regions of the SE-US, also within the *AoU* Secure Cloud Computing Platform. The Grocery Gap Atlas was used to incorporate the Retail Food Environment Index (RFEI) and related localized food access indicators, allowing for a standardized assessment of the availability of healthy versus energy-dense food outlets [29,30]. These environmental metrics were further contextualized using Feeding America’s Map the Meal Gap data, which provided county-level estimates of FI prevalence, poverty rates, and the relative cost of food [18]. To account for the divergent impacts of rural versus urban landscapes, participants were categorized using USDA Rural-Urban Commuting Area (RUCA) codes, facilitating a classification system that distinguishes the logistical challenges of rurality from the metabolic risks associated with processed-food dense urban environments [30]. Table 2 summarizes demographic, socioeconomic, and geographic characteristics of the analytic cohort and is intended to characterize the study population rather than provide population-representative estimates for the Southeastern United States.

### 2.4. Privacy Compliance and Statistical Controls

Given the sensitivity of residential data, specific geospatial aggregation procedures were employed to ensure participant privacy and compliance with *AoU* security protocols.

Residential information, originally derived from participant-reported ZIP codes, was aggregated to the ZIP3 level (the first three digits of the ZIP code) according to the HIPAA Privacy Rule Safe Harbor deidentification standard (45 CFR §164.514[b][2]). This procedure masks specific household locations while maintaining sufficient granular detail to link neighborhood-level deprivation indices and environmental characteristics to each participant [31]. Finally, to account for unmeasured regional variation, including healthcare infrastructure, and sun exposure, state of residence was identified and included in all regression models as a fixed effect [32]. While these approaches protected participant confidentiality and enabled linkage to contextual environmental measures, they may have introduced geographic misclassification by obscuring local variation in neighborhood conditions. Additionally, the adjusted models may not fully account for within-state heterogeneity in food environments, healthcare access, and socioeconomic conditions. Given these limitations, this hierarchical approach ensures that the observed biomarker differences associated with FI are analyzed within the appropriate structural and geographic frameworks [32].

### 2.5. Biomarker Categorization and Outcomes

This study examined a panel of nutritional, metabolic, and inflammatory biomarkers measured only in pregnant participants at enrollment as part of routine clinical laboratory testing [33]. Metabolic and inflammatory biomarkers of interest included: Body Mass Index (BMI), High-Density Lipoprotein (HDL), Low-Density Lipoprotein (LDL), Systolic Blood Pressure (SBP), Diastolic Blood Pressure (DBP), non-fasting glucose, and C-reactive protein (CRP). Nutritional biomarkers included ferritin, Vitamin B12, Vitamin D (25-hydroxyvitamin D), folate, and albumin due to their relevance to micronutrient status, protein status, inflammation, and metabolic health during pregnancy [34,35,36]. All biomarkers were analyzed at enrollment as continuous outcomes to preserve variability and avoid information loss associated with arbitrary categorization [37]. However, because biomarkers were obtained at the time of assessment, they may be influenced by gestational age, plurality, and pregnancy-related physiological changes.

Given these constraints, biomarker outcomes were assessed through a dual-panel approach, evaluating nutritional status, inflammation, and cardiometabolic health. The metabolic panel included high-sensitivity CRP, HDL, LDL, SBP, DBP, and non-fasting glucose levels, reflecting systemic inflammation and cardiometabolic status. In parallel, nutritional status was characterized through a panel of biomarkers reflecting micronutrient reserves, protein status, and inflammatory regulation, specifically serum albumin, Vitamin B12, Vitamin D, folate, and ferritin. To enhance clinical relevance, these continuous biomarker data were harmonized to reduce extreme outliers without removing important physiological data. This approach may facilitate the identification of clinically meaningful thresholds, offering insights that could support the development of targeted maternal health interventions in vulnerable populations [38].

### 2.6. Urbanicity

Urbanicity was classified using established 2022 RUCA codes aggregated at the ZIP3 level. Urbanicity measures were used descriptively and in stratified analyses [39,40].

### 2.7. Regression Framework and Geographic Stratification

Multivariable linear regression models were used to evaluate associations between food insecurity and biomarker outcomes. These models were selected because the primary outcomes (nutritional, metabolic, and inflammatory biomarkers) were continuous variables. These models were used to estimate adjusted associations between food insecurity and biomarker concentrations while accounting for individual-level and neighborhood-level covariates. Neighborhood deprivation and environmental indices were incorporated to capture broader contextual factors associated with food access, socioeconomic disadvantages, and the environment that may influence maternal health. Continuous biomarker variables were winsorized at the 1st and 99th percentiles to reduce the influence of extreme observations while preserving the overall distribution of the data. Sensitivity analyses were conducted by refitting all primary regression models using the original non-winsorized biomarker values. Results were compared to assess whether substantive conclusions were influenced by extreme observations (Supplementary Table S1).

Separate models were fit for each biomarker, with FI status as the primary exposure variable. Models adjusted for the available demographic and area-level socioeconomic covariates, including ethnicity, median household income, county-level poverty, neighborhood deprivation index, and urbanicity. To evaluate whether associations between FI and biomarker outcomes differed by geographic context, an interaction term between FI and urbanicity (FI × urbanicity) was included in each model. Biomarker-specific complete-case analyses were performed because laboratory data availability varied across outcomes. Biomarker-specific complete-case analyses were performed, resulting in varying sample sizes across outcomes due to differences in data availability. For interaction analyses, regression coefficients (β), 95% confidence intervals (CIs), and *p*-values were reported. To account for multiple testing across biomarker outcomes, Benjamini–Hochberg false discovery rate (FDR) correction was applied to interaction-model *p*-values. Statistical significance was defined as a two-sided *p*-value < 0.05.

$$Y_{i}=\beta_{0}+\beta_{1}\left({FI}_{i}\right)+\beta_{2}(Urbanicity)+\beta_{3}({FI}_{i}\times {Urbanicity}_{i})+\sum_{k}\gamma \kappa X_{\kappa i}+\varepsilon_{i}$$

where $Y_{i}$ represents the specific winsorized biomarker outcome for individual biomarkers. Utilizing the winsorized continuous biomarkers (_wins) eliminated the need for log-transformations, allowing coefficients to be interpreted directly on their native clinical scales. ${FI}_{i}$ represents individual-level FI status, ${Urbanicity}_{i}$ denotes a binary indicator (Urban vs. Rural), and $X_{\kappa i}$ represents a vector of individual-level demographic and socioeconomic covariates. To estimate associations independent of key sociodemographic factors, models were adjusted for ethnicity, median household income, county-level poverty, neighborhood deprivation index, and urbanicity. Several pregnancy-related variables, including parity, smoking status, prenatal vitamin use, gestational age, pre-pregnancy BMI, and pregnancy-related comorbidities, were unavailable or incompletely captured and therefore could not be included in the adjustment strategy. Continuous predictors were mean-centered and standardized to a standard deviation of 1.0 to facilitate direct comparison of effect sizes.

Given the number of biomarkers evaluated, FDR correction was applied to interaction-model *p*-values to account for multiple comparisons. Both unadjusted and FDR-adjusted *p*-values are reported.

Models were adjusted for ethnicity, median household income, county-level poverty, neighborhood deprivation index, and urbanicity (α_state_). Furthermore, because individuals within the same sub-state geographic boundaries share overlapping environmental exposures, robust standard errors clustered at the Zip3 level were deployed to correct for spatial autocorrelation and avoid inflation of Type I error rates [41,42]. Following the global interaction testing, fully adjusted stratified models comparing urban versus rural contexts were executed. This stratification was critical for identifying how the biological consequences of FI are either amplified or mitigated by the specific constraints of the local landscape, such as transportation barriers in rural areas versus the prevalence of highly processed, energy-dense foods in urban centers [43].

### 2.8. Environmental and Food Landscape Indicators

The analytical regression models incorporated neighborhood-level environmental indicators and state-fixed effects to account for the structural context of participant residences. FI status, demographic characteristics, and socioeconomic variables including self-reported median income, Area Deprivation Index (ADI), Social Vulnerability Index (SVI), and SNAP participation were obtained from participant-completed *AoU* surveys. Nutritional and inflammatory biomarkers were obtained from EHRs and physical measurement data collected at or near enrollment. Neighborhood deprivation, poverty, transportation access, food environment characteristics, and urbanicity measures were obtained from external geographic datasets and linked to participants using ZIP3-level residential identifiers. These validated datasets included poverty rates and transportation access, derived from established geographic and social vulnerability frameworks [39,44]. Poverty rates were based on the 2023 American Community Survey estimates with data collected between 2019 and 2023 and, within the 2022 Map the Meal Gapand 2022 Grocery Gap Atlas (1997–2023) frameworks, were integrated with participant-completed survey measures of median income and SNAP participation to characterize financial barriers to food access. Transportation access and geographic isolation were assessed using 2022 RUCA classifications and the proportion of households lacking private vehicle access, capturing infrastructural constraints that may limit food acquisition. Broader structural disadvantage was characterized using validated deprivation metrics, including ADI and SVI, which aggregate multiple census-level indicators of socioeconomic status, household composition, and housing quality.

To further characterize the nutritional landscape, the models incorporated food environment measures, including the RFEI and food desert severity metrics from the Grocery Gap Atlas [29]. Collectively, these measures enabled the analysis to distinguish between financial hardship, transportation-related barriers, and broader neighborhood disinvestment, providing insight into how individual-level FI and area-level contextual factors may jointly relate to variation in nutritional and metabolic biomarkers within resource-constrained or processed-food-dominant environments [17,45]. Derivation of RFEI below:

$$RFEI=\frac{\left(Fast\;Food\;Restaurants+Convenience\;Stores\right)}{\left(Supermarkets+Farmers\;Markets\right)}$$

Although efforts were made to use geographic datasets corresponding as closely as possible to the period of participant enrollment, neighborhood measures may not have necessarily been collected contemporaneously with survey responses or biomarker assessments. Therefore, integrated analyses may be subject to temporal misalignment and residual measurement error.

### 2.9. Data Harmonization and Quality Control

Data extraction and cleaning were performed across multiple data subsets to build a unified cohort for analytic modeling. Prior to merging, variables spanning clinical and demographic domains were systematically evaluated for structural consistency and data integrity.

A multi-step data harmonization pipeline was implemented to address technical inconsistencies across disparate relational datasets. Participant records were integrated using a unique identifier (*person_id*). To prevent type-mismatch errors during dataset linkage, all identifiers were standardized as character strings, ensuring consistent string matching and preservation of leading alphanumeric elements.

Geographic filtering was subsequently applied to derive a cohesive analytical cohort. Participants lacking a valid state designation, including zero-length entries or missing/unmappable state metadata, were excluded.

To ensure structural integrity of the analytical dataset, laboratory and EHR data were evaluated for participant-level duplication. Duplicate records were resolved using a first-occurrence selection approach, retaining a single unique observation per confirmed pregnant individual and preserving a one-to-one correspondence with the master cohort. This approach minimized the risk of artificial sample size inflation and bias in downstream analyses.

Finally, to reduce the influence of extreme values and potential data-entry anomalies in clinical and laboratory measures, symmetric winsorization was applied to all continuous metabolic and nutritional biomarkers. Values for BMI, HDL, LDL, SBP, DBP, non-fasting glucose, and CRP below the 1st percentile or above the 99th percentile were set to the corresponding percentile thresholds. The frequency and percentage of biomarker values affected by winsorization and the full winsorized analysis can be found in Supplemental Tables S2 and S1, respectively. This approach limited the impact of outliers while preserving the full sample and maintaining the integrity of the original measurement scales, thereby improving the stability and interpretability of subsequent analyses.

### 2.10. Statistical Analysis and Complete-Case Profiling

Consistent with the biomarker classification described in Section 2.5, descriptive and comparative analyses were organized into two clinically relevant domains: a metabolic/inflammatory panel and a nutritional panel. Descriptive and comparative baselines were established by stratifying clinical profiles across two distinct thematic paradigms: a Metabolic Table (comprising BMI, HDL, LDL, SBP, DBP, non-fasting Glucose, and CRP) and a Nutritional Table (comprising VitaminB12, Albumin, Vitamin D3, Folate, and Ferritin).

Due to continuous laboratory variables exhibiting differential missingness patterns inherent to real-world clinical data, summary tables were generated using independent complete-case frameworks rather than whole-cohort deletion. This approach maximized statistical power within each analytic domain by retaining participants with available data while avoiding unnecessary exclusion due to missing variables in unrelated domains. However, laboratory data in EHR-based cohorts are often not missing at random, as test ordering is influenced by clinical need and provider behavior. As a result, complete-case analyses may introduce selection bias, and findings should be interpreted considering potential systematic differences between individuals with and without observed laboratory measurements. Continuous variables are expressed as mean ± standard deviation (SD) unless otherwise specified. These analyses were considered descriptive comparisons and utilized ANOVA, Chi-squared, Student’s *t*-test and non-parametric testing to determine statistical significance (*p* < 0.05).

All data manipulation, harmonization, deduplication pipelines, and automated table generation were executed within the secure *AoU* R Statistical Computing Environment. [Tidyverse] core principles were maintained via the [dplyr] package for programmatic dataset mutations, column structural drops, and relational [left_join] cohorts, while complete-case baseline clinical characteristics were aggregated and formatted using the [tableone] package.

### 2.11. Ethics and Data Governance

The *AoU* Research Program protocol was approved by a centralized institutional review board, and all participants provided informed consent and were de-identified [33]. All analyses were conducted in compliance with the *AoU* Data User Code of Conduct and Data and Statistics Dissemination Policy. No participant-level data were removed from the Researcher Workbench, and all reported results adhere to required cell-size suppression thresholds to protect participant privacy [33].

Race and ethnicity were treated as social constructs reflecting differential exposure to structural and environmental determinants of health. Adjustment was performed to reduce confounding, with results interpreted cautiously to avoid masking health disparities [46].

## 3. Results

### 3.1. Geospatial Distribution and Study Cohort

**Figure 1:**
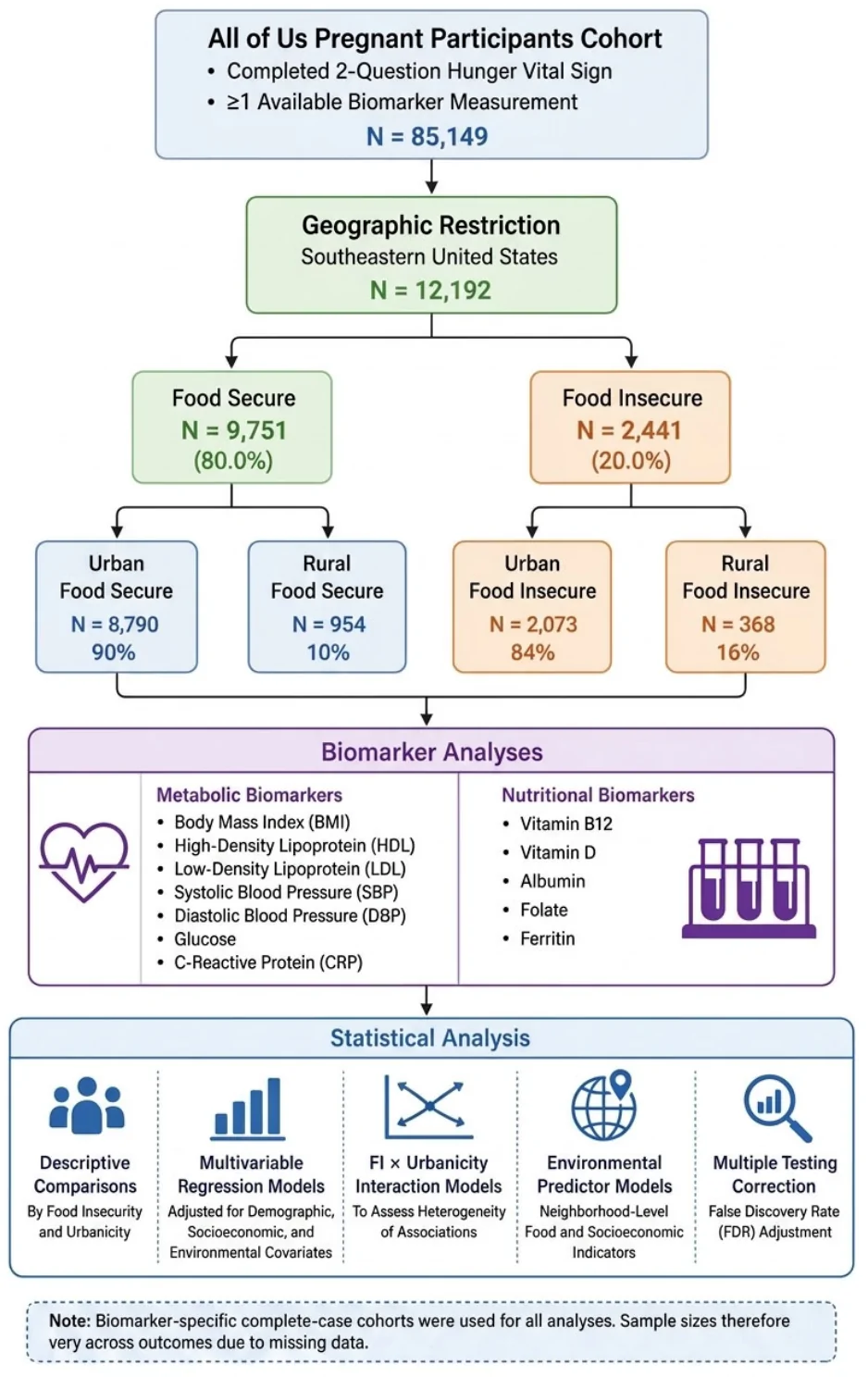
Study population derivation and analytic workflow.

The initial phase of the analysis focused on the spatial distribution of the study population across the Southeastern United States. Utilizing a robust GIS and statistical modeling framework, this study then mapped the prevalence of food insecurity (FI) while maintaining strict adherence to privacy protocols (Figure 1).

The following is an overview of the study population, geographic classification, and analytic approach. The initial *AoU* Cohort Builder query identified 85,149 pregnant participants who completed the FI assessment and had at least one eligible biomarker measurement available. Geographic restriction to the Southeastern United States yielded a final analytic cohort of 12,192 participants. Among these, 9751 (80%) were classified as food-secure and 2441 (20%) as food-insecure before undergoing descriptive, biomarker-specific, and multivariable regression analyses. Biomarker-specific complete-case cohorts were winsorized and used for all laboratory analyses, resulting in varying sample sizes across outcomes. GIS modeling using ZIP3 data and RUCA classifications was used to stratify participants into urban and rural environments. Nutritional, metabolic, and inflammatory biomarkers measured at enrollment were analyzed using multivariable regression models adjusted for ethnicity, median household income, county-level poverty, neighborhood deprivation index, and urbanicity, to evaluate associations between FI, environmental context, and biomarker outcomes.

A total of 12,192 pregnant participants residing in the SE-US met inclusion criteria for this analysis (Table 1). Among these, 9751 (80%) were classified as food-secure and 2441 (20%) as food-insecure based on the 2-item FI screening instrument [28]. ZIP3-level residential data were linked with RUCA codes to evaluate whether associations between FI and maternal biomarkers differed across geographic contexts. This analysis generated four analytic subgroups, urban food-secure, rural food-secure, urban food-insecure, and rural food-insecure, that were then analyzed by either metabolic or nutritional biomarkers. FI differed significantly by urbanicity. A greater proportion of food-insecure participants resided in rural areas compared with food-secure participants (16% vs. 10%, *p* < 0.001). Among the 2441 food-insecure participants included in the analytic cohort, 2073 (84%) resided in urban settings and 368 (16%) resided in rural settings. In contrast, among food-secure participants, a smaller proportion resided in rural areas, indicating a relative overrepresentation of rural residence within the food-insecure group (Figure 1).

**Table 1:** Demographic, Clinical, and Socioeconomic Characteristics of Pregnant Participants Stratified by Food Security Status.

Demographic	Food-Secure (*n*, %)	Food-Insecure (*n*, %)	*p*-Value
**Sample Size**	9751	2441	
**Age in years (mean ± SD)**	33.4 ± 7.19	33.7 ± 7.12	0.6282
**Gestational Age (weeks)** **(mean ± SD)**	32.3 ± 11.5	31.1 ± 10.5	0.9824
**Race**			***0.004***
Asian/Native Hawaiian/Pacific Islander	247 (2)	25 (1)	
Black or African America	1609 (17)	709 (29)	
White/Middle Eastern/North African	6624 (68)	1358 (56)	
More than one population	222 (2)	73 (3)	
Other/I Prefer Not to Answer	1052 (11)	276 (11)	
**Ethnicity**			0.169
Hispanic or Latino	1185 (12)	292 (12)	
Not Hispanic or Latino	8404 (86)	2088 (86)	
Other/I Prefer Not to Answer	161 (2)	61 (2)	
**Highest Education Level**			***<0.001***
Advanced Degree	3414 (35)	247 (10)	
College Graduate	2885 (30)	470 (20)	
Some College	2303 (24)	960 (40)	
High School Diploma/GED	802 (8)	554 (22)	
Less Than High School	190 (2)	147 (6)	
Prefer Not to Answer	156 (1)	63 (2)	
**Annual Income**			***<0.001***
≤35k	1984 (20)	1357 (56)	
35k–75k	2410 (25)	605 (25)	
75k–150k+	2598 (44)	194 (7)	
Prefer Not to Answer	1067 (11)	285 (12)	
**Employment Status**			***<0.001***
Employed/Self-Employed	6118 (57)	1193 (43)	
Student	768 (8)	165 (7)	
Unemployed/Unable to work/Homemaker	1341(14)	926 (38)	
Prefer Not to Answer/Skip/NA	1523 (16)	157 (6)	
**Health Insurance**			***<0.001***
Yes	9054 (93)	1964 (80)	
No	553 (6)	386 (16)	
Don’t Know/Prefer Not to Answer/Skip	143 (1)	91 (4)	
**Current Marital Status**			***<0.001***
Married/Living with Partner	5617 (58)	995 (40)	
Divorced/Separated/Widowed	1689 (17)	632 (26)	
Never Married	2328 (24)	751 (31)	
Prefer Not to Answer/Skip	116 (1)	63 (3)	

Notes: Values are presented as *n* (%) unless otherwise indicated. Percentages are calculated within food security status. Sample sizes may vary across variables due to missing responses. Responses categorized as “Prefer not to answer,” “Don’t know,” or missing are displayed where applicable. Counts fewer than 20 are suppressed in accordance with *AoU* Research Program data dissemination policies. *p*-values are indicative of associations between Food-Insecure and Food-Secure cohorts and represented in bold and italics (*p* < 0.05). Wilcoxon rank sum tests (WRSTs) were used for continuous variables; χ^2^ tests were used for categorical variables.

Additionally, area-level characteristics are presented in Table 2. Spatial and housing characteristics differed significantly between food-secure and food-insecure participants. Geographic distribution varied across Southeastern states (*p* < 0.001). Food-insecure participants were disproportionately represented in Alabama (32% vs. 27%), Mississippi (6% vs. 4%), South Carolina (6% vs. 4%), and Tennessee (11% vs. 8%), whereas food-secure participants were more frequently represented in Georgia (16% vs. 10%), Florida (24% vs. 21%), and Virginia (6% vs. 3%).

Significant differences were also observed in urbanicity (*p* < 0.001). Food-insecure participants were more likely to reside in rural areas compared with food-secure participants (16% vs. 10%), while a greater proportion of food-secure participants resided in urban areas (90% vs. 84%). These findings suggest a higher burden of FI among rural populations within the Southeastern United States.

Housing characteristics further differed by food security status (*p* = 0.004). Although single-family homes and townhouses were the predominant housing type in both groups, food-insecure participants were less likely to reside in these housing types compared with food-secure participants (89% vs. 92%). Food-insecure participants were also more likely to report an unknown or missing housing type (5% vs. 2%).

**Table 2:** Geographic and Housing Characteristics of Pregnant Participants Stratified by Food Security Status.

Spatial Characteristics	Food-Secure (*n*, %)	Food-Insecure (*n*, %)	*p*-Value
**Sample Size**	9751	2441	
**State**			***<0.001***
Alabama (AL)	2679 (27)	783 (32)	
Florida (FL)	2292 (24)	502 (21)	
Georgia (GA)	1534 (16)	256 (10)	
Kentucky (KY)	206 (2)	80 (3)	
Mississippi (MS)	413 (4)	136 (6)	
North Carolina (NC)	749 (8)	161 (7)	
South Carolina (SC)	436 (4)	140 (6)	
Tennessee (TN)	733 (8)	269 (11)	
Virginia (VA)	613 (6)	80 (3)	
West Virginia (WV)	95 (1)	34 (1)	
**Urbanicity**			***<0.001***
Rural	954 (10)	368 (16)	
Urban	8796 (90)	2073 (84)	
**Main Type of Housing**			***0.004***
Single-family home/townhouse	8997 (92)	2166 (89)	
Apartments/condominium	524 (6)	156 (6)	
Don’t Know/Skip	229 (2)	119 (5)	

Notes: Values are presented as *n* (%) unless otherwise indicated. Percentages are calculated within food security status. Sample sizes may vary across variables due to missing responses. Responses categorized as “Prefer not to answer,” “Don’t know,” or missing are displayed where applicable. Counts fewer than 20 are suppressed in accordance with *All of Us* Research Program data dissemination policies. *p*-values are indicative of associations between Food-Insecure and Food-Secure cohorts and represented in bold and italics (*p* < 0.05). Wilcoxon rank sum tests (WRSTs) were used for continuous variables; χ^2^ tests were used for categorical variables.

**Figure 2:**
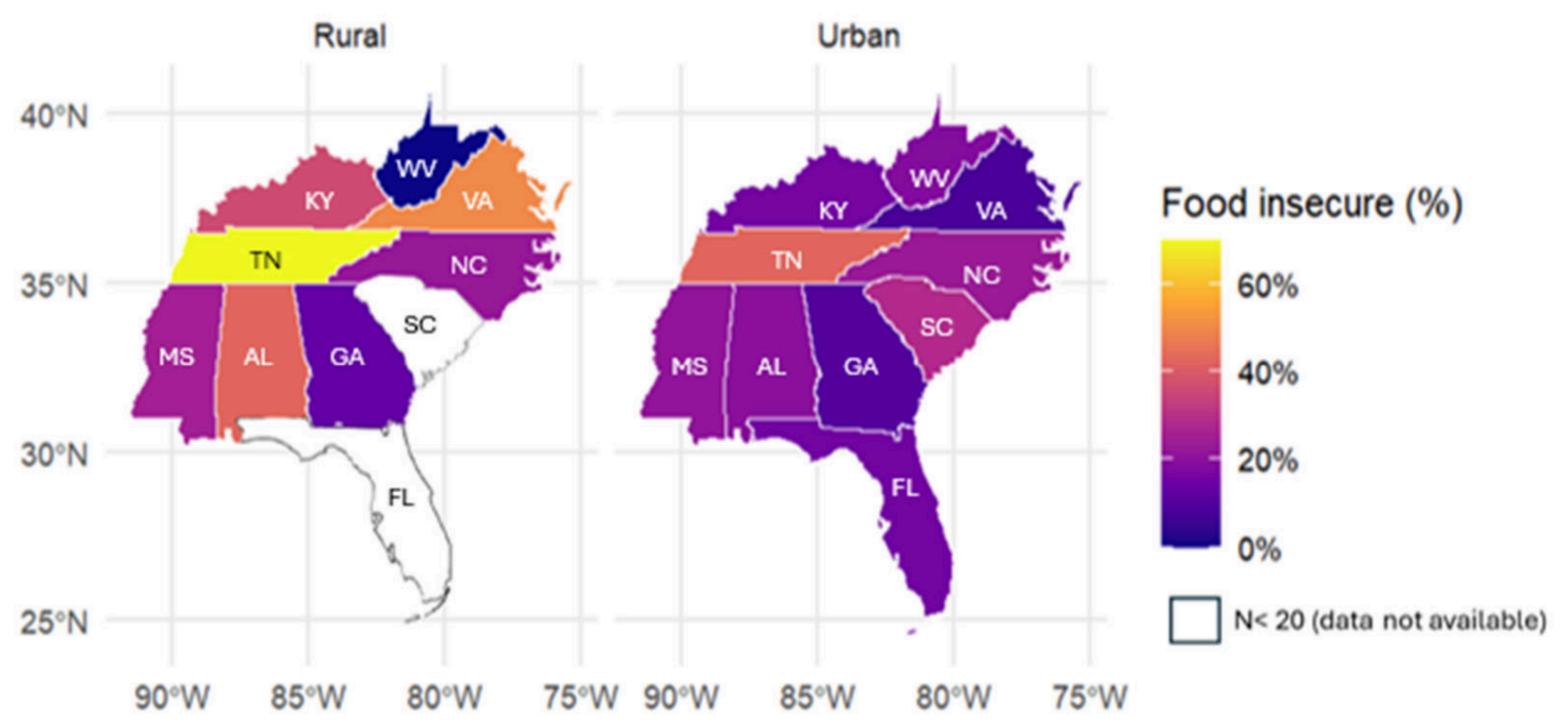
Geographic distribution of FI data across the Southeastern United States.

Geographic visualization identified substantial heterogeneity in FI prevalence across the SE-US. Rural participants consistently demonstrated a higher prevalence of FI compared with their urban counterparts across most states. Tennessee demonstrated one of the highest proportions of FI among pregnant participants within the SE-US *AoU* cohort. States shaded in the final analysis represent those with sufficient ZIP3-level data (*N* > 20), ensuring the statistical power necessary to evaluate regional fixed effects (Figure 2). State-specific prevalence estimates are provided in Supplemental Table S3.

FI status was derived from participant self-reported responses to the Hunger Vital Sign survey administered through the *AoU* Research Program. Values represent the distribution of participants within the study cohort and are not intended to provide population-representative estimates of FI prevalence at the state level. Figure 2 shows the geographic distribution of FI among pregnant participants enrolled in the *AoU* Research Program across Southeastern U.S. states, stratified by rural and urban residence using RUCA classification. Color intensity represents the percentage of participants classified as food-insecure within each state-specific rural or urban subgroup based on the 2-item FI screening instrument. Higher values (yellow) indicate greater prevalence of FI. White shading indicates states with fewer than 20 participants in a subgroup, which were suppressed due to data availability and privacy considerations.

### 3.2. Metabolic Profiles by Food Security Status

Following data harmonization, deduplication, and quality control procedures, analytic datasets were constructed using biomarker-specific complete-case frameworks. This approach resulted in variable sample sizes across analyses due to differential data availability. Cardiometabolic indicators were evaluated within the metabolic analytic cohort, defined as participants with available laboratory or clinical measurements for each respective outcome (Table 3).

In the overall cohort, food-insecure participants exhibited a higher mean BMI compared with food-secure participants (33.59 ± 9.40 vs. 29.47 ± 7.80 kg/m^2^; *p* < 0.001). HDL cholesterol was lower among food-insecure individuals (51.94 ± 15.55 vs. 57.17 ± 14.11 mg/dL; *p* = 0.001), while LDL was modestly higher (110.69 ± 29.79 vs. 106.78 ± 27.68 mg/dL; *p* = 0.044). DBP was also higher among food-insecure participants (76.35 ± 6.49 vs. 74.82 ± 7.20 mmHg; *p* = 0.029), whereas SBP did not differ significantly between groups (*p* = 0.145). Non-fasting serum glucose concentrations were elevated among food-insecure individuals (125.16 ± 37.04 vs. 117.84 ± 26.61 mg/dL; *p* < 0.001). CRP levels were higher in the food-insecure group (5.0 [4.8, 6.32] vs. 3.1 [3.0, 4.0] mg/L; *p* = 0.001).

### 3.3. Associations of Food Security Status and Urbanicity with Cardiometabolic Biomarkers

Stratified analyses were conducted within urban and rural populations using harmonized complete-case datasets (Table 4). Sample sizes varied across strata due to the combined requirements of biomarker availability and mappable geographic data.

BMI remained significantly higher among food-insecure participants in both urban and rural settings (*p* < 0.001 for both comparisons). In urban areas, mean BMI was 33.38 ± 9.37 kg/m^2^ among food-insecure individuals compared with 29.24 ± 7.66 kg/m^2^ among food-secure individuals. In rural settings, corresponding values were 34.76 ± 9.48 kg/m^2^ and 31.71 ± 8.79 kg/m^2^. Non-fasting serum glucose concentrations were higher among food-insecure participants in urban settings (124.57 ± 36.56 vs. 117.45 ± 26.15 mg/dL; *p* < 0.001), whereas no significant differences were observed in rural populations (*p* = 0.697). Across both food security groups, mean glucose levels were generally higher in rural participants compared with their urban counterparts. For lipid measures, HDL was lower among food-insecure participants in urban areas (52.38 ± 15.69 vs. 57.30 ± 15.80 mg/dL; *p* < 0.001), while this difference did not reach statistical significance in rural settings (*p* = 0.054). LDL and SBP did not differ significantly by food security status in either geographic context (all *p* > 0.05). DBP was modestly higher among food-insecure participants in urban settings (76.45 ± 6.52 vs. 74.76 ± 7.17 mmHg; *p* = 0.017), but not in rural populations (*p* = 0.235). Median CRP levels did not differ significantly by food security status within either urban or rural strata (*p* = 0.079 and *p* = 0.933, respectively), despite higher absolute values observed among urban food-insecure participants.

### 3.4. Nutritional Biomarkers by Food Security Status

Nutritional biomarkers were analyzed using a separate complete-case framework restricted to participants with available laboratory values for each measure (Table 5).

Food-insecure participants had lower mean concentrations of several key micronutrients, including vitamin B12 (505.57 ± 344.23 vs. 565.91 ± 331.61 pg/mL; *p* = 0.006), albumin (37.11 ± 12.68 vs. 39.40 ± 9.98 g/L; *p* < 0.001), vitamin D (28.36 ± 12.92 vs. 33.64 ± 13.36 ng/mL; *p* < 0.001), and folate (12.18 ± 6.22 vs. 14.82 ± 6.57 ng/mL; *p* < 0.001). Ferritin concentrations did not differ significantly between food security groups (median 42.50 [17.22–89.88] vs. 38.30 [18.56–81.08] ng/mL; *p* = 0.306).

### 3.5. Nutritional Biomarker Profiles by Food Security Status and Urbanicity

Stratified analyses of nutritional biomarkers by urbanicity are summarized in Table 6. Sample sizes varied across strata due to differential completeness of biomarker measurements.

In urban populations, food-insecure participants had lower concentrations of vitamin B12 (515.61 ± 345.74 vs. 573.92 ± 327.44 pg/mL; *p* < 0.001), albumin (37.93 ± 11.68 vs. 39.90 ± 9.05 g/L; *p* < 0.001), vitamin D (28.11 ± 12.84 vs. 33.69 ± 13.34 ng/mL; *p* < 0.001), and folate (12.41 ± 5.96 vs. 15.14 ± 6.90 ng/mL; *p* < 0.001) compared with food-secure participants.

In contrast, no statistically significant differences by food security status were observed in rural populations for vitamin B12 (*p* = 0.877), albumin (*p* = 0.67), vitamin D (*p* = 0.743), or folate (*p* = 0.986). Across both food security groups, rural participants generally exhibited lower mean albumin and folate levels compared with urban participants. Ferritin concentrations were not significantly associated with food security status in either urban (*p* = 0.064) or rural settings (*p* = 0.698).

**Figure 3:**
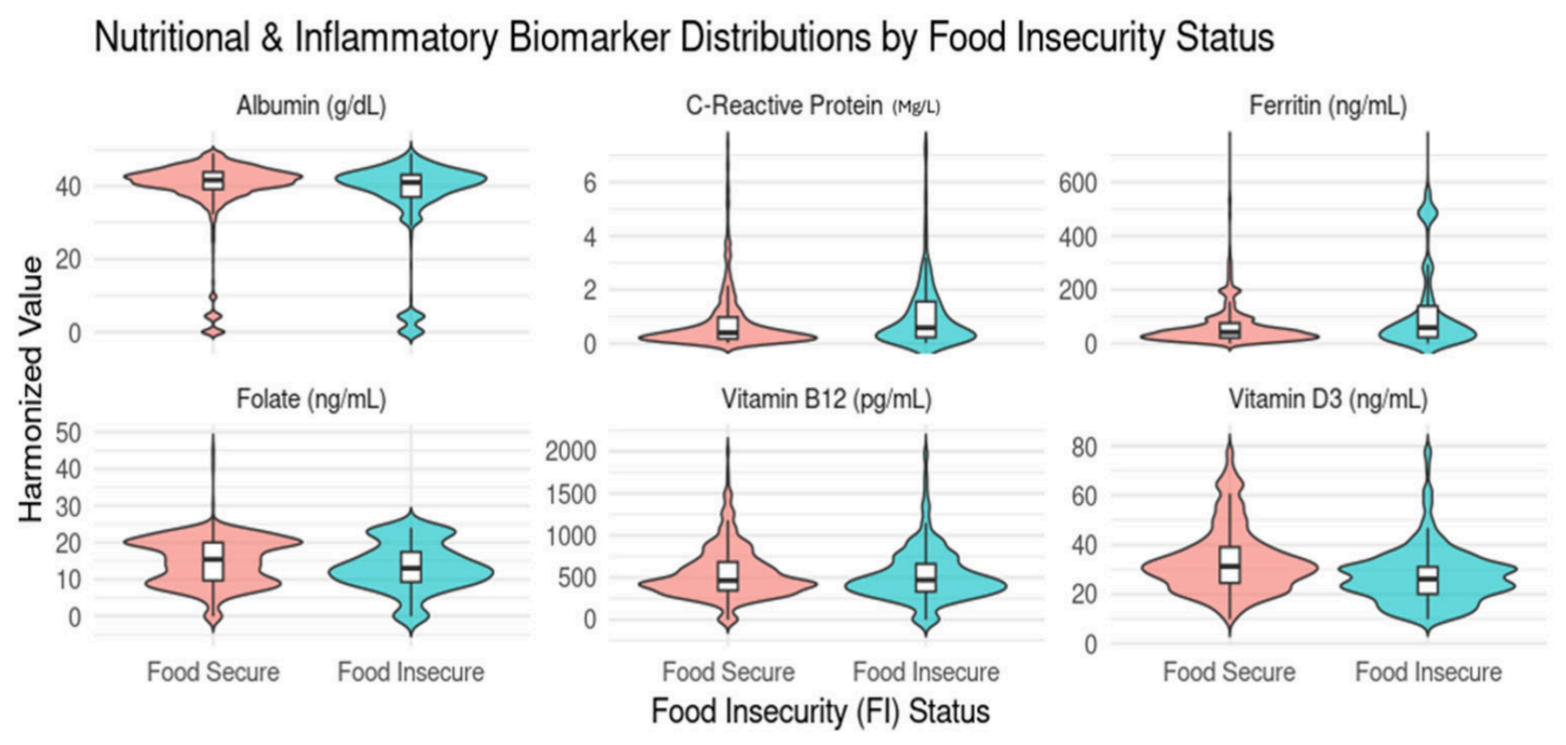
Distributions of nutritional and inflammatory biomarkers by food security status.

Complementing these results, Figure 3 presents the distributions of key nutritional and inflammatory biomarkers by food security status, providing a descriptive overview of variability across groups.

Figure 3 illustrates the distributions of key nutritional and inflammatory biomarkers by food security status. Overall, the violin plots show broadly similar distributions between food-secure and food-insecure groups, with some modest shifts in central tendency and variability. Albumin levels appear tightly clustered in both groups with minimal differences in median values. In contrast, CRP and ferritin exhibit slightly higher central values and greater dispersion among food-insecure participants.

Violin plots illustrate the distribution of biomarker values among food-secure and food-insecure participants, with overlaid boxplots indicating medians and interquartile ranges. Biomarkers include albumin, CRP, ferritin, folate, Vitamin B12, and Vitamin D3. Values are derived from complete-case analytic samples for each biomarker and were winsorized (Supplemental Table S2). These distributions are presented for descriptive comparison (Food-Secure: 9712, Food-Insecure: 2432).

For micronutrient markers, food-insecure individuals show modestly lower median concentrations of folate and vitamin D3 compared to food-secure individuals, with somewhat wider variability. Vitamin B12 distributions are largely overlapping across groups, although the food-insecure group displays a slightly broader range of values. Ferritin distributions also overlap substantially, despite greater spread among food-insecure participants.

### 3.6. Evaluation of Food Insecurity × Urbanicity Interactions

Formal interaction analyses were conducted to evaluate whether associations differed by urbanicity. FI was significantly associated with BMI, HDL cholesterol, vitamin D, and folate concentrations; however, none of the FI × urbanicity interaction terms remained statistically significant after FDR correction for multiple comparisons. These findings suggest that observed urban–rural differences are more consistent with geographic heterogeneity in associations than statistically significant effect modification (Table 7).

To formally evaluate whether associations differed by urbanicity between FI and biomarker outcomes, interaction models were fit for each biomarker including FI status, urbanicity, and a FI × urbanicity interaction term (Table 7). FI was independently associated with higher BMI (β = 3.47, *p* < 0.001) and glucose concentrations (β = 11.68, *p* = 0.002), as well as lower HDL cholesterol (β = −3.21, *p* = 0.001), vitamin D (β = −4.61, *p* = 0.007), and folate concentrations (β = −1.92, *p* = 0.01). Albumin, vitamin B12, ferritin, CRP, systolic blood pressure, diastolic blood pressure, and LDL cholesterol were not significantly associated with FI after adjustment. Although a nominally significant interaction was observed for folate (interaction β = −5.24, 95% CI: −10.13 to −0.35, *p* = 0.03), this association did not remain significant following false discovery rate correction (FDR-adjusted *p* = 0.42). No other FI × urbanicity interaction terms were statistically significant (all FDR-adjusted *p* > 0.05). These findings suggest that while FI was associated with several nutritional and metabolic biomarkers, the magnitude of these associations did not differ significantly between rural and urban participants. The adjusted R^2^ values were generally modest across biomarker models, indicating that although FI and the included socioeconomic and geographic covariates explained a portion of biomarker variability, a substantial proportion of variation remained unexplained. This finding is consistent with the multifactorial nature of maternal nutritional, inflammatory, and metabolic health and suggests that no single environmental or socioeconomic factor fully accounts for observed biomarker differences. Even though only a limited number of biomarkers demonstrated statistically significant associations, these results showed that statistically significant main-effect associations should be interpreted as nominal associations and not as findings confirmed following multiple-comparison adjustment.

Sensitivity analyses comparing models fitted using original and winsorized biomarker values yielded generally consistent findings across biomarkers (Supplemental Table S1). For HDL cholesterol, glucose, vitamin D, folate, albumin, LDL cholesterol, CRP, vitamin B12, ferritin, systolic blood pressure, and diastolic blood pressure, effect estimates, confidence intervals, and statistical inference were largely unchanged following winsorization. The greatest differences were observed for BMI. Examination of the EHR-derived data identified several implausibly high BMI values, including values exceeding 200 kg/m^2^ and multiple values exceeding 3000 kg/m^2^. These observations substantially influenced the non-winsorized model and obscured the underlying association between FI and BMI. Winsorization reduced the influence of these extreme values, resulting in a more stable and interpretable estimate. Following winsorization, the association between FI and BMI became more stable and statistically significant, suggesting that extreme observations obscured the underlying association in the original data. Overall, the findings indicate that the primary associations were not driven by a small number of extreme observations.

To determine whether these associations differed significantly between urban and rural populations, formal FI × urbanicity interaction models were evaluated for each biomarker. Although FI was independently associated with several biomarkers, no interaction terms remained statistically significant after FDR correction, suggesting that the magnitude of these associations was generally comparable across geographic settings. However, exploratory stratified analyses identified different patterns of association between environmental factors and biomarker concentrations across urban and rural models (Table 8). In rural settings, multiple predictors were significantly associated with markers of nutritional status, whereas fewer significant associations were observed in urban environments.

### 3.7. Divergent Environmental Predictors: Rural vs. Urban Landscapes

The final stage of the analysis characterized how the local food environment differentially impacts maternal biomarkers in rural versus urban settings using regression models. In rural areas, higher poverty rates and greater exposure to food swamp environments (measured using the RFEI) were associated with lower albumin and vitamin B12 concentrations, suggesting that structural and environmental conditions may be related to variation in markers of nutritional status. To further characterize contextual variability, analyses were stratified by urbanicity among all participants (Table 8) and adjusted for state-fixed effects. In rural areas, several environmental and structural indicators were associated with nutritional biomarkers. Higher county-level poverty and greater retail food environment imbalance (higher RFEI scores) were associated with lower albumin and Vitamin B12 levels, while greater vehicle access showed positive associations with these biomarkers.

Exploratory stratified analyses identified different patterns of association between environmental factors and biomarker concentrations across urban and rural models (Table 8). In rural settings, multiple predictors were significantly associated with markers of nutritional status, whereas fewer significant associations were observed in urban environments.

For albumin, a marker of protein nutritional status, higher county-level vehicle access was positively associated with albumin concentrations in rural pregnant participants (β = +6.09, *p* < 0.001), while no association was observed in urban residents. In contrast, adverse food retail environments were associated with lower albumin levels in rural settings, as reflected by a negative association with food swamp index (β = −0.48, *p* < 0.01). Higher county poverty was also strongly associated with lower albumin concentrations in rural populations (β = −3.54, *p* < 0.001), indicating reduced nutritional reserves in areas of greater structural economic deprivation.

Similar rural-specific patterns were observed for Vitamin B12. Greater vehicle access was strongly associated with higher Vitamin B12 levels in rural adults (β = 136.31, *p* < 0.001), while increased county-level poverty (β = −59.47, *p* < 0.001) and higher food swamp exposure (β = −14.36, *p* < 0.05) were associated with lower Vitamin B12 concentrations. None of these predictors demonstrated significant associations with Vitamin B12 levels among urban participants.

In contrast to albumin and Vitamin B12, ferritin showed an urban-specific association. Higher food swamp exposure was positively associated with ferritin levels in urban adults (β = +0.07, *p* < 0.05), while no significant relationship was detected in rural populations.

For HDL, vehicle access was associated with lower HDL levels across both rural (β = −5.41, *p* < 0.05) and urban settings (β = −1.74, *p* < 0.05), indicating a consistent relationship between transportation access and cardiometabolic markers regardless of residence status.

Folate concentrations were also evaluated because of their importance in maternal and fetal health. In both rural and urban models, folate was not significantly associated with food environment measures, poverty, vehicle access, or BMI.

## 4. Discussion

The present study examined associations between self-reported household food insecurity (FI), maternal nutritional, metabolic, and inflammatory biomarkers, and geographic context among pregnant participants residing in the Southeastern United States. By integrating participant-reported FI, clinical biomarkers, and neighborhood-level environmental indicators, this analysis expands current understanding of how FI may be associated with measurable variation in maternal health during pregnancy. Several observed associations, including higher BMI, higher non-fasting glucose concentrations, lower HDL cholesterol, and lower concentrations of selected nutritional biomarkers among food-insecure participants, were consistent with findings from prior research. In addition, the integration of geospatial and environmental measures provided an opportunity to examine whether these associations differed across urban and rural settings. Although stratified analyses suggested geographic variation in several associations, formal FI × urbanicity interaction testing provided limited evidence that these associations differed significantly by urbanicity after adjustment for multiple comparisons. Accordingly, the observed geographic differences should be interpreted as exploratory heterogeneity in associations rather than evidence of true effect modification. Given the cross-sectional design and residual potential for unmeasured confounding, these findings should be interpreted as adjusted associations and hypothesis-generating observations rather than evidence of causal relationships.

Consistent with prior research, geographic visualization revealed heterogeneity in FI prevalence across the southeastern United States, with higher rates among rural participants compared with urban populations in several states [43,47,48]. These patterns are consistent with evidence that FI is shaped by local economic conditions, transportation access, food availability, and other community-level factors [49]. While the geographic clustering of FI is consistent with previous studies, our study links geographic patterns to measured maternal nutritional, inflammatory, and metabolic biomarkers. This approach extends prior work by examining potential biological associations of self-reported FI within a large and geographically diverse pregnant population.

### 4.1. Geographic Context of Food Insecurity and Biomarker Patterns

Geographic visualization of FI across the Southeastern United States further highlights important contextual differences between rural and urban environments (Figure 2). In this figure, rural regions demonstrate greater heterogeneity in FI prevalence across states, with certain areas, such as Tennessee and Virginia, exhibiting notably higher percentages compared to neighboring regions. In contrast, urban FI appears more spatially uniform, with moderate prevalence distributed across most metropolitan areas. Notably, some states (Tennessee and Kentucky) show relatively elevated FI rates in both rural and urban contexts, suggesting broader regional influences beyond urbanicity alone. These patterns indicate that FI is not only unevenly distributed geographically but may also reflect distinct structural and environmental conditions across settings. These findings are consistent with the literature indicating that FI and food access are shaped by local economic conditions, geographic distance to food retailers, household vehicle availability, and the broader food environment [50]. The greater variability observed in rural areas may reflect localized resource constraints, including transportation barriers and limited food retail access, whereas the more consistent urban distribution may reflect shared characteristics of urban food environments, such as widespread availability of energy-dense, low-cost foods [49]. Together, these findings reinforce the importance of geographic context in shaping FI exposure and provide additional support for the observed differences in biomarker associations across rural and urban populations.

Another key contribution of this study is the identification of differences in how FI is associated with biomarkers across these established regions. However, these differences should be interpreted as exploratory and hypothesis-generating rather than definitive biological patterns [14,47].

### 4.2. Micronutrient Status and Hidden Nutritional Vulnerability

Cardiometabolic differences observed in the overall cohort suggest that recent self-reported household FI was associated with several metabolic biomarkers. Food-insecure individuals had higher BMI and elevated non-fasting glucose concentrations than food-secure participants (Table 3).

Food-insecure participants also exhibited lower HDL cholesterol, modestly higher LDL cholesterol, and higher DBP, although not all cardiometabolic markers differed consistently across settings (Table 3). This pattern is broadly consistent with prior studies linking FI to dyslipidemia and cardiovascular risk factors, particularly among women [51,52]. Differences in CRP were observed in the overall cohort, with higher median levels among food-insecure individuals (Table 3). However, these differences were not statistically significant within stratified urban and rural analyses. Previous population-based studies have reported associations between FI, elevated CRP, and other markers of systemic inflammation [9,11]. The absence of statistically significant differences in the stratified analyses may reflect smaller sample size or differences in the FI-CRP association across settings. Given the cross-sectional design and potential influence of adiposity, these findings should be interpreted as associative rather than causal.

In urban populations, FI was consistently associated with higher BMI, elevated non-fasting glucose, lower HDL cholesterol, higher DBP, and lower concentrations of vitamin B12, vitamin D, albumin, and folate (Table 4 and Table 6). These findings suggest that in urban environments, FI in pregnant participants may be associated with a combination of metabolic strain and worse nutritional status [14]. The coexistence of elevated BMI and reduced micronutrient levels is consistent with prior descriptions of dietary patterns characterized by energy-dense but nutrient-poor food intake [53].

In rural populations, fewer statistically significant differences in biomarkers were observed by FI status (Table 4 and Table 6). While BMI remained higher among food-insecure participants, differences in glucose, lipid markers, inflammatory measures, and nutritional biomarkers were not statistically significant. Rural populations generally had lower albumin and folate concentrations than urban participants, regardless of FI status [54,55]. These findings suggest that broader structural constraints beyond individual FI classification may contribute to nutritional differences across rural and urban settings.

In all, these results suggest that the associations between FI and biomarkers may differ by geographic context; however, it is important to note that they do not establish distinct causal biological pathways. Instead, they highlight the importance of considering both individual-level and contextual factors when evaluating maternal nutritional and metabolic health.

Across the full cohort, FI was associated with lower concentrations of several key nutritional biomarkers, including vitamin B12, albumin, vitamin D, and folate (Table 5). These findings suggest that recent self-reported household FI may be associated with differences in micronutrient-related and nutritional biomarkers across multiple domains. Such patterns are consistent with prior work demonstrating that FI is linked to poor diet quality and reduced intake of nutrient-dense foods [4,56]. Vitamin B12, vitamin D, and folate are important during pregnancy because they contribute to maternal nutritional status and have recognized roles in fetal growth and neurodevelopment. [57,58]. In stratified analyses, lower vitamin B12, vitamin D, albumin, and folate concentrations were observed among FI urban participants, whereas these differences were not statistically significant among rural participants. The absence of statistically significant rural differences should not be interpreted as evidence that rural FI does not affect nutritional status, but rather that the smaller rural sample size and possible differences in demographic and clinical characteristics may have limited ability to detect associations within this subgroup.

Albumin, evaluated here as an integrated marker of protein status, inflammation, and systemic health, further highlights the cumulative physiological burden imposed by rural FI on pregnant women [59]. In contrast, ferritin concentrations did not differ significantly by FI status in the overall cohort, suggesting that iron status may be maintained despite broader nutritional vulnerability. This may reflect dietary patterns that preserve certain nutrients through fortification or supplementation, even in the presence of overall dietary inadequacy [60,61].

### 4.3. Environmental Contexts and Biomarker Influence

Formal interaction testing provided limited evidence that associations differed by urbanicity after adjustment. Although stratified analyses identified geographic differences in several biomarkers, the absence of significant interaction effects after correction for multiple comparisons suggests that these findings likely reflect contextual variation across settings rather than statistically significant effect modification by urbanicity (Table 7).

Exploratory stratified analyses identified different patterns of association across urban and rural models (Table 8). These findings should be interpreted cautiously because formal FI × urbanicity interaction testing did not identify statistically significant differences after false discovery rate correction. Accordingly, the observed patterns are best interpreted as exploratory contextual variation rather than evidence of effect modification by urbanicity. In rural settings, multiple adjusted biomarkers, including albumin and vitamin B12, were strongly associated with structural and environmental factors such as vehicle access, food environment quality, and county-level poverty. Higher vehicle access was associated with increased albumin and vitamin B12 levels, suggesting that transportation plays a critical role in facilitating access to nutritionally adequate foods, particularly those rich in protein and animal-source nutrients. This aligns with prior work showing that rural residents often face longer distances to food retailers and rely heavily on transportation to access diverse, nutrient-dense food sources [62].

Conversely, higher county poverty and poorer retail food environments (as measured by RFEI) were associated with lower albumin and vitamin B12 levels in rural settings (Table 8). These findings suggest that adverse environmental and socioeconomic conditions in rural settings were associated with lower concentrations of selected nutritional biomarkers [63]. Albumin, a marker of protein status and longer-term nutritional reserves, may reflect chronic dietary inadequacy in these settings, while vitamin B12, largely obtained from animal-source foods, may be particularly sensitive to reduced availability of nutrient-dense foods in disadvantaged rural environments [64,65]. Taken together, these findings suggest that transportation access, economic deprivation, and food environment characteristics may be associated with variation in nutritional biomarkers within rural settings.

In contrast, the urban findings were more limited but reveal distinct patterns, particularly for ferritin. While most structural factors were not significantly associated with albumin or vitamin B12 in urban settings, higher RFEI (more “food swamp” environments) was associated with increased ferritin levels. This counterintuitive pattern suggests that urban FI may reflect a different nutritional and metabolic profile, characterized less by overt micronutrient depletion and more by metabolic dysregulation [18,53].

One possible explanation is that urban food environments, even when dominated by energy-dense and low-quality foods, often include fortified products or processed foods that contain iron. As a result, iron status may be preserved, or even elevated, despite overall dietary inadequacy in pregnant women [66]. Additionally, ferritin is not only a marker of iron stores but also an acute-phase reactant, meaning that elevated levels may reflect underlying inflammation rather than improved nutritional status [66]. Thus, in urban FI contexts, higher ferritin may represent a combination of dietary exposures and inflammation-related processes, rather than optimal nutrient intake.

This divergence between biomarkers highlights the importance of distinguishing between different dimensions of nutritional status. The observed differences between rural and urban stratified models should be interpreted cautiously because FI × urbanicity interaction terms were not statistically significant after FDR correction. Additional studies incorporating dietary intake, healthcare access, and other environmental factors are needed to clarify the mechanisms underlying these associations.

Notably, vehicle access was negatively associated with HDL cholesterol in both rural and urban settings. This consistent relationship suggests that transportation access may capture broader lifestyle or environmental factors, such as reduced reliance on active transportation or increased access to processed and convenience foods. While access to transportation may improve food acquisition in rural settings, it may simultaneously be associated with dietary patterns that adversely affect lipid profiles across both contexts. This highlights the complexity of interpreting structural determinants: the same factor may confer both benefits and risks, depending on the pathway considered. Because the study did not assess physical activity, transportation mode, dietary intake, or food purchasing patterns, the mechanisms underlying this relationship cannot be determined. Taken together, these findings indicate that area-level economic, transportation, and food-environment indicators were associated with selected maternal biomarkers, particularly in rural settings.

Although associations involving folate and ferritin did not consistently achieve statistical significance, both biomarkers demonstrated notable variation across food security and geographic strata. Folate findings are noteworthy despite the lack of consistent statistical significance because folate is a critical micronutrient during pregnancy and fetal neural tube development [35,67]. Similarly, ferritin findings warrant careful interpretation because ferritin reflects both iron stores and inflammatory processes [68,69]. Consequently, ferritin concentrations may not exclusively represent nutritional status and should be interpreted within a broader physiological context. Folate concentrations tended to be lower among food-insecure participants and varied by urbanicity, whereas ferritin demonstrated substantial heterogeneity across groups. Given the established role of folate and iron status in maternal and fetal health, these findings may warrant further investigation in larger cohorts with more complete biomarker ascertainment.

### 4.4. Environmental Context and Biomarker Associations

The observed associations between FI, geography, and biomarker profiles support the idea that structural and environmental contexts may shape how FI is reflected biologically. However, given the cross-sectional design and observational nature of the data, these findings should be interpreted as associative rather than causal.

The persistence of associations between FI and several biomarkers after adjustment for socioeconomic indicators suggests that FI captures dimensions of vulnerability not fully explained by traditional socioeconomic measures alone. At the same time, the variability in findings across urban and rural settings highlights the possibility that broader structural conditions, such as food environments, transportation access, and healthcare access, may contribute to heterogeneity in these associations for pregnant women [18,70].

Several findings confirmed expected associations between FI and adverse nutritional or metabolic profiles, including higher BMI and lower HDL cholesterol. However, other findings, particularly the variability observed across geographic contexts, suggest that structural and environmental factors may contribute to these relationships in ways not fully captured by individual-level measures alone [71]. Notably, formal FI × urbanicity interaction analyses did not remain statistically significant after FDR correction, indicating that observed urban–rural differences should be interpreted as geographic heterogeneity in associations rather than evidence of true effect modification. These findings may inform future studies evaluating transportation access, food environments, and other structural factors as potential intervention targets, although intervention effectiveness cannot be evaluated within the present study. The relatively modest adjusted R^2^ values observed across models further suggest that maternal nutritional and metabolic status is influenced by numerous biological, behavioral, clinical, and environmental factors that were not fully captured within the current dataset. Consequently, the observed associations should be viewed as one component of a broader and more complex set of determinants affecting maternal health.

Overall, the present study both confirms previously reported associations between FI and maternal nutritional and metabolic biomarkers and contributes new evidence regarding the geographic and environmental context in which these associations occur. While biomarker findings reflect maternal physiological status rather than direct measures of fetal health, they highlight potential pathways through which socioeconomic and environmental adversity may influence maternal well-being during pregnancy. Future longitudinal studies incorporating maternal and neonatal outcomes will be necessary to evaluate temporality and potential downstream effects on infant health.

### 4.5. Limitations

Several limitations should be considered when interpreting these findings. First, the cross-sectional design precludes assessment of temporality and prevents causal inference. Consequently, the observed associations between FI, environmental exposures, and biomarker profiles should be interpreted as associative rather than causal. Additionally, despite the identification of statistically significant differences across several biomarkers, the magnitude of some effects was modest; therefore, statistical significance should not be equated with clinical significance.

Sensitivity analyses demonstrated that the majority of observed associations were robust to winsorization. However, BMI results were sensitive to a small number of implausible EHR-derived values, highlighting the importance of addressing extreme observations when analyzing routinely collected clinical data.

Residual confounding also remains possible. Although models adjusted for key demographic, socioeconomic, and environmental factors, several pregnancy-relevant variables were not consistently available within the dataset, including parity, prenatal vitamin use, dietary intake, smoking or vaping behaviors, pre-pregnancy BMI, and pregnancy-related comorbidities. These unmeasured factors may have influenced both FI status and biomarker concentrations.

The use of EHR-derived laboratory data introduces additional challenges. Laboratory testing in clinical settings is influenced by provider practices, healthcare utilization, and clinical indication; therefore, missing laboratory values are unlikely to be missing completely at random. Biomarker-specific complete-case analyses may have introduced selection bias and contributed to differences in sample size across outcomes. This limitation is particularly relevant for biomarkers such as folate, ferritin, and vitamin B12, which were available only for subsets of participants.

Several limitations are also associated with the integration of multiple data sources. FI was determined from participant self-reported responses to the Hunger Vital Sign questionnaire and was not independently verified, introducing the potential for recall bias, reporting bias, and exposure misclassification. Furthermore, neighborhood-level indicators were derived from external datasets collected across overlapping but not identical time periods. As a result, some degree of temporal misalignment between survey responses, biomarker measurements, and environmental exposures may have occurred.

An additional consideration is that participant enrollment and FI assessments occurred during a period that overlapped with the COVID-19 pandemic. Pandemic-related disruptions in employment, food availability, healthcare utilization, and social assistance programs may have influenced both reported household FI and health-related outcomes. Because the present study was not designed to specifically evaluate pandemic-related effects, the potential influence of COVID-19-related social and economic disruptions cannot be excluded.

Geographic and environmental measures were assigned using ZIP3-level residential identifiers to comply with *All of Us* privacy requirements. Although this approach enabled linkage to contextual measures while protecting participant confidentiality, it may have obscured important local variation in food environments, transportation infrastructure, healthcare access, and socioeconomic conditions. Similarly, composite indices such as the ADI and SVI provide useful summaries of contextual disadvantage but may mask variability in individual components and may not fully capture participants’ lived experiences.

Finally, the *AoU* Research Program is a voluntary research cohort rather than a population-based probability sample. Although the cohort is more diverse than many traditional biomedical research populations, participants may differ from the broader Southeastern U.S. population in demographic, socioeconomic, and healthcare-related characteristics. Therefore, findings may not be fully generalizable to all pregnant individuals in the SE-US.

Importantly, this study evaluated maternal nutritional, metabolic, and inflammatory biomarkers rather than pregnancy or neonatal outcomes. While several biomarkers examined are relevant to fetal growth and development, the present analysis cannot determine whether the observed maternal biomarker differences translate into adverse neonatal outcomes. Future longitudinal studies incorporating both maternal and infant outcomes are needed to clarify these relationships.

### 4.6. Strengths

Despite these limitations, this study has several notable strengths. The analysis leverages a large, diverse, pregnancy-relevant subset of the *AoU* Research Program, enabling the examination of FI across a heterogeneous population. The integration of survey responses, electronic health record-derived clinical measures, laboratory biomarkers, and geospatial data provides a multidimensional perspective on maternal health. Importantly, the inclusion of nutritional, metabolic, and inflammatory biomarkers extends the investigation of FI beyond self-reported measures, allowing for the identification of potential biological correlates of FI during pregnancy. Additionally, the linkage of individual-level data with rural–urban classifications and food-environment indicators facilitates the examination of context-specific patterns and highlights the importance of place-based influences on maternal health. The regional focus on the SE-US is particularly relevant given the persistent socioeconomic, nutritional, and healthcare inequities that characterize many communities within this region. Although the findings should be interpreted as exploratory and hypothesis-generating, they provide an important foundation for future multilevel investigations examining how individual, environmental, and structural factors jointly shape maternal nutritional and metabolic outcomes during pregnancy.

Future research should prioritize longitudinal designs with repeated assessment of nutritional and metabolic biomarkers across pregnancy to identify potential windows of vulnerability and resilience. Building on the findings presented here, future studies should incorporate multilevel environmental measures alongside clinical, behavioral, genomic, epigenetic, and inflammatory data to better characterize the pathways through which FI and broader social determinants of health may become biologically embedded. Such efforts will be critical for informing targeted, context-specific interventions aimed at reducing maternal and neonatal health disparities, particularly in regions of the United States that experience a disproportionate burden of FI and structural disadvantage.

## 5. Conclusions

This study suggests that food insecurity (FI) may function not only as a social or economic indicator, but also as a factor associated with measurable biological variation in maternal health. By integrating individual-level biomarkers from the *AoU* Research Program with geospatial and environmental indicators, this analysis identifies patterns indicating that the relationship between FI and nutritional and metabolic biomarkers may differ across physical environments. Specifically, the findings highlight associations between individual food access, neighborhood-level deprivation, and variation in micronutrient status and metabolic markers.

The observed differences between rural and urban cohorts represent an important contribution to the literature on maternal health disparities. Although exploratory stratified analyses suggested geographic variation in associations between FI and selected biomarkers, formal interaction testing did not identify statistically significant urban–rural differences after false discovery rate correction. Accordingly, these findings should be interpreted as hypothesis-generating rather than evidence of effect modification. Exploratory stratified analyses suggested that urban environments may exhibit patterns consistent with relatively stable micronutrient markers alongside evidence of metabolic strain; however, these observations require confirmation in future studies [61]. While these interpretations are consistent with existing hypotheses, the underlying biological and behavioral mechanisms require further investigation.

These findings have implications for research, policy, and clinical practice. From a research perspective, future longitudinal studies are needed to clarify temporal relationships between FI, nutritional status, and pregnancy outcomes. From a policy perspective, the results support continued investment in food assistance programs, transportation access initiatives, and community-level interventions designed to improve access to nutrient-dense foods in underserved areas. Clinically, routine FI screening during pregnancy may help identify individuals who could benefit from nutritional counseling, targeted micronutrient supplementation, food assistance referrals, or enhanced prenatal monitoring.

The strengths of this study include the large and geographically diverse cohort, integration of biomarker and environmental data, and evaluation of both nutritional and metabolic outcomes. However, the cross-sectional design, reliance on self-reported FI, biomarker-specific missingness, and use of area-level environmental indicators limit causal interpretation and generalizability. Accordingly, findings should be interpreted as associations rather than causal effects.

Ultimately, these results suggest that improving maternal nutrition may require approaches that address both individual FI and the broader structural conditions that influence access to healthy foods. Such efforts may support more equitable maternal health outcomes and inform the development of geographically tailored nutritional interventions during pregnancy.

## Figures and Tables

**Table 3:** Unadjusted cardiometabolic indicators by food security status in pregnant participants.

Metabolic Indicators	Food-Secure	Food-Insecure	*p*-Value
***N***	9712	2432	
**BMI (kg/m^2^)**	29.47 ± 7.80	33.59 ± 9.40	***<0.001***
**HDL Cholesterol (mg/dL)**	57.17 ± 14.11	51.94 ± 15.55	***0.001***
**LDL Cholesterol (mg/dL)**	106.78 ± 27.68	110.69 ± 29.79	***0.044***
**Systolic Blood Pressure (mmHg)**	124.74 ± 12.35	126.48 ± 11.21	0.145
**Diastolic Blood Pressure (mmHg)**	74.82 ± 7.20	76.35 ± 6.49	***0.029***
**Serum Glucose (non-fasting) (mg/dL)**	117.84 ± 26.61	125.16 ± 37.04	***<0.001***
**C-Reactive Protein (mg/L)**	3.1 [3.0, 4.0]	5.0 [4.8, 6.32]	***0.001***

Note: All values are mean ± SD unless otherwise stated. Non-normally distributed are represented as median [IQR]. The final analytical sample for nutritional and metabolic variables may vary across the evaluated biomarkers due to incomplete laboratory assays and the exclusion of participants missing baseline food security data. *p*-values in bold and in italics denote significance.

**Table 4:** Cardiometabolic biomarkers by food security status and urbanicity.

	Urban			Rural		
	Food-Secure	Food-Insecure	*p*	Food-Secure	Food-Insecure	*p*
***N***	8758	2064		954	368	
**BMI (kg/m^2^)**	29.24 ± 7.66	33.38 ± 9.37	***<0.001***	31.71 ± 8.79	34.76 ± 9.48	***<0.001***
**HDL Cholesterol (mg/dL)**	57.30 ± 15.80	52.38 ± 15.69	***<0.001***	54.16 ± 18.24	48.37 ± 14.01	0.054
**LDL Cholesterol (mg/dL)**	106.90 ± 27.60	110.17 ± 29.28	0.1	102.38 ± 30.51	118.59 ± 37.03	0.107
**Systolic Blood Pressure (mmHg)**	122.16 ± 7.13	124.69 ± 12.38	0.118	128.14 ± 9.95	122.16 ± 7.13	0.365
**Diastolic Blood Pressure (mmHg)**	74.76 ± 7.17	76.45 ± 6.52	***0.017***	78.51 ± 8.36	72.10 ± 3.04	0.235
**Serum Glucose (non-fasting) (mg/dL)**	117.45 ± 26.15	124.57 ± 36.56	***<0.001***	126.76 ± 34.67	130.06 ± 41.15	*0.697*
**C-Reactive Protein (mg/L)**	3.1 [3.0, 4.0]	5.4 [5.20, 6.72]	0.079	4.3 [4.18, 6.37]	4.5 [4.30, 5.74]	0.933

Note: All values are mean ± SD unless otherwise stated. Non-normally distributed are represented as median [IQR]. The final analytical sample for nutritional and metabolic variables varies across the evaluated biomarkers due to incomplete laboratory assays and the exclusion of participants missing baseline food security data. *p*-values that are bolded and in italics denote significance (*p* < 0.05).

**Table 5:** Nutritional biomarker concentrations by food security status in pregnant participants.

Outcome	Food-Secure	Food-Insecure	*p*-Value
***N***	9712	2432	
**Vitamin B12 (pg/mL)**	565.91 ± 331.61	505.57 ± 344.23	***0.006***
**Albumin (g/L)**	39.40 ± 9.98	37.11 ± 12.68	***<0.001***
**Vitamin D (ng/mL)**	33.64 ± 13.36	28.36 ± 12.92	***<0.001***
**Folate (ng/mL)**	14.82 ± 6.57	12.18 ± 6.22	***<0.001***
**Ferritin (ng/mL)**	38.30 [18.56, 81.08]	42.50 [17.22, 89.88]	0.306

Note: All values are mean ± SD unless otherwise stated. Non-normally distributed are represented as median [IQR]. The final analytical sample for nutritional and metabolic variables may vary across the evaluated biomarkers due to incomplete laboratory assays and the exclusion of participants missing baseline food security data. *p*-values that are bolded and in italics denote significance (*p* < 0.05).

**Table 6:** Nutritional biomarkers by food security status and urbanicity.

	Urban			Rural		
	Food-Secure	Food-Insecure	*p*	Food-Secure	Food-Insecure	*p*
***N***	8758	2064		954	368	
**Vitamin B12 (pg/mL)**	573.92 ± 327.44	515.61 ± 345.74	***<0.001***	420.13 ± 374.17	552.74 ± 328.17	0.877
**Albumin (g/L)**	39.90 ± 9.05	37.93 ± 11.68	***<0.001***	30.17 ± 18.38	31.19 ± 17.25	0.67
**Vitamin D (ng/mL)**	33.69 ± 13.34	28.11 ± 12.84	***<0.001***	31.96 ± 14.06	30.69 ± 13.70	0.743
**Folate (ng/mL)**	15.14 ± 6.9	12.41 ± 5.96	***<0.001***	10.94 ± 10.94	10.91 ± 9.42	0.986
**Ferritin (ng/mL)**	38.83 [19.00, 80.43]	42.00 [17.00, 90.00]	0.064	29.43 [8.00, 88.50]	61.95 [19.40, 77.50]	0.698

**Note:** All values are mean ± SD unless otherwise stated. Non-normally distributed are represented as median [IQR]. The final analytical sample for the nutritional variables may vary across the evaluated biomarkers due to incomplete laboratory assays and the exclusion of participants missing baseline food security data. *p*-values that are bolded and in italics denote significance (*p* < 0.05).

**Table 7:** Interaction Between Food Insecurity and Urbanicity in Association With Biomarker Outcomes.

Biomarker	*N*	Adj_R^2^	FI β	FI *p*-Value	Interaction β	Interaction LCL	Interaction UCL	Interaction *p*-Value	Interaction FDR
**BMI (kg/m^2^)**	12,144	0.04	3.47	***p* < 0.001**	−0.49	−2.83	1.83	0.67	0.86
**HDL Cholesterol (mg/dL)**		0.04	−3.21	**0.001**	1.36	−3.86	6.60	0.60	0.86
**LDL Cholesterol (mg/dL)**		0.01	−2.18	0.59	−6.51	−22.32	9.28	0.41	0.86
**Systolic Blood Pressure (mmHg)**		0.08	3.69	0.14	2.31	−9.69	14.31	0.70	0.86
**Diastolic Blood Pressure (mmHg)**		0.01	1.76	0.33	0.06	−8.60	8.73	0.98	0.98
**Serum Glucose (non-fasting) (mg/dL)**		0.02	11.68	**0.002**	−2.43	−20.38	15.50	0.78	0.86
**Albumin (g/L)**		0.002	−0.59	0.33	−0.98	−4.00	2.04	0.52	0.86
**Vitamin B12 (pg/mL)**		−1.27 × 10^−5^	19.72	0.58	30.76	−188.37	249.89	0.78	0.86
**Vitamin D (ng/mL)**		0.02	−4.61	**0.007**	−10.92	−29.58	7.73	0.25	0.86
**Folate (ng/mL)**		0.04	−1.92	**0.01**	−5.24	−10.13	−0.35	**0.03**	0.42
**Ferritin (ng/mL)**		0.01	−9.42	0.58	47.81	−37.56	133.19	0.27	0.86
**C-Reactive Protein (mg/L)**		0.04	2.86	0.40	−4.60	−17.90	8.70	0.49	0.86

Notes: LCL—Lower Confidence Limit; UCL—Upper Confidence Limit; FDR—False Discovery Rate.

**Table 8:** Exploratory Urban- and Rural-Specific Associations Between Environmental Factors and Biomarker Outcomes.

Biomarker	Urbanicity	Predictor	Beta	Std Error	*p*-Value	Adjusted R^2^
Albumin (g/dL)	Rural	RFEI (Food Swamp)	*−0.48*	*0.17*	*0.005*	0.64
		County Poverty (%)	*−3.54*	*0.32*	*<0.001*	
		No Vehicle Access (%)	*6.10*	*0.92*	*<0.001*	
		BMI	*−0.22*	*0.10*	*0.027*	
	Urban	RFEI (Food Swamp)	−0.23	0.14	0.115	0.02
		County Poverty (%)	−0.13	0.14	0.369	
		No Vehicle Access (%)	0.06	0.58	0.923	
		BMI	*−0.13*	*0.05*	*0.003*	
Vitamin B12 (pg/mL)	Rural	RFEI (Food Swamp)	*−14.36*	*6.51*	*0.035*	0.35
		County Poverty (%)	*−59.47*	*15.00*	*<0.001*	
		No Vehicle Access (%)	*136.31*	*46.41*	*0.006*	
		BMI	*−8.37*	*4.11*	*0.050*	
	Urban	RFEI (Food Swamp)	1.51	5.83	0.796	−0.01
		County Poverty (%)	−3.22	7.24	0.657	
		No Vehicle Access (%)	−17.24	24.52	0.483	
		BMI	−1.22	1.94	0.529	
Folate (ng/mL)	Rural	RFEI (Food Swamp)	0.03	0.18	0.877	−0.06
		County Poverty (%)	−0.40	0.55	0.473	
		No Vehicle Access (%)	1.34	1.38	0.346	
		BMI	−0.13	0.11	0.285	
	Urban	RFEI (Food Swamp)	0.25	0.23	0.261	−0.02
		County Poverty (%)	0.28	0.23	0.261	
		No Vehicle Access (%)	−0.20	1.03	0.846	
		BMI	0.03	0.85	0.708	
Ferritin (ng/mL)	Rural	RFEI (Food Swamp)	0.05	1.74	0.111	−0.07
		County Poverty (%)	−0.04	0.10	0.683	
		No Vehicle Access (%)	0.28	0.22	0.216	
		BMI	0.00	0.02	0.804	
	Urban	RFEI (Food Swamp)	*0.07*	*0.03*	*0.016*	0.01
		County Poverty (%)	0.02	0.02	0.352	
		No Vehicle Access (%)	−0.17	0.11	0.120	
		BMI	0.00	0.01	0.657	
HDL (mg/Hg)	Rural	RFEI (Food Swamp)	0.05	0.25	0.849	0.15
		County Poverty (%)	1.04	0.80	0.202	
		*No Vehicle Access* (*%*)	*−5.41*	*2.14*	*0.015*	
		*BMI*	*−0.34*	*0.17*	*0.046*	
	Urban	RFEI (Food Swamp)	−0.02	0.21	0.926	0.06
		County Poverty (%)	0.36	0.20	0.065	
		*No Vehicle Access* (*%*)	*−1.74*	*0.82*	*0.035*	
		*BMI*	*−0.32*	*0.06*	*<0.001*	

Notes: Estimates represent associations between environmental or structural factors and biomarker levels, stratified by urbanicity, with corresponding interpretations provided for significant findings.

## Data Availability

This study used data from the *All of Us* Research Program’s Controlled Tier Dataset (version 7) available to authorized users on the Researcher Workbench.

## Supplementary Materials

The following supporting information can be downloaded at https://www.mdpi.com/article/10.3390/nu18182965/s1, Table S1: Comparison of Regression Coefficients Before and After Winsorization. Table S2: Frequency and Percentage of Biomarker Values Affected by Winsorization. Table S3: State-Specific Prevalence of Food Insecurity by Urbanicity Among Pregnant Participants in the Southeastern United States.

## Abbreviations

The following abbreviations are used in this manuscript:

*AoU*	*All of Us* Research Program
SE-US	Census-Designated Southeastern United States
EHR	Electronic Health Records
FI	Food Insecurity
