# Supplementary material for: Geographic and Environmental Differences in Associations Between Food Insecurity and Nutritional and Inflammatory Biomarkers Among Pregnant Participants in the All of Us Research Program
Source: Nutrients. 2026 Sep 10;18(18):2965. doi: 10.3390/nu18182965 (PMC13610282; doi:10.3390/nu18182965)
Supplement: Supplementary file 1 [file nutrients-18-02965-s001.zip › nutrients-4483116-supplementary.pdf]

Supplemental Table S1: Comparison of Regression Coefficients Before and After Winsorization

Biomarker	Original β	Original LCL	Original UCL	Original P-value	Winsorized β	Winsorized LCL	Winsorized UCL	Winsorized P- value
BMI	-0.282	-10.917	10.353	0.959	3.473	2.595	4.35	*<0.001*
HDL	-3.245	-5.334	-1.157	0.002	-3.219	-5.245	-1.193	0.002
LDL	-2.014	-10.44	6.411	0.639	-2.188	-10.25	5.873	0.594
Glucose	12.004	4.032	19.975	0.003	11.684	4.161	19.207	0.002
CRP	1.835	-6.122	9.793	0.65	2.865	-3.843	9.572	0.401
Ferritin	-185.318	-443.315	72.68	0.159	-9.426	-42.928	24.076	0.581
Vitamin B12	16.294	-60.384	92.973	0.676	19.725	-51.224	90.675	0.585
Vitamin D	-4.413	-7.927	-0.899	0.014	-4.616	-8.011	-1.222	0.008
Folate	-2.076	-3.654	-0.499	0.01	-1.928	-3.447	-0.41	0.013
SBP	3.553	-1.666	8.773	0.181	3.696	-1.312	8.703	0.147
DBP	1.719	-2.131	5.569	0.38	1.768	-1.839	5.375	0.335
Albumin	-0.591	-1.798	0.615	0.337	-0.58	-1.779	0.618	0.342

Supplemental Table S2: Frequency and Percentage of Biomarker Values Affected by Winsorization

Biomarker	Overall Cohort (*N*)	Winsorized (*N*)	Winsorized (%)
BMI	12144	247	2.04
HDL		210	1.73
LDL		264	2.18
Glucose		252	2.08
CRP		134	1.11
Ferritin		265	2.19
Vitamin B12		289	2.38
Vitamin D		252	2.08
Folate		274	2.26
SBP		230	1.9
DBP		190	1.57

Supplemental Table S3: State-Specific Prevalence of Food Insecurity by Urbanicity Among Pregnant Participants in the Southeastern United States

	Urban (%)		Rural (%)	
	**Food Secure**	**Food Insecure**	**Food Secure**	**Food Insecure**
**Alabama (AL)**	80.28	19.72	57.14	42.86
**Florida (FL)**	84.40	15.60	≤ 20	≤ 20
**Georgia (GA)**	89.49	10.51	86.96	13.04
**Kentucky (KY)**	84.00	16.00	63.64	36.36
**Mississippi (MS)**	78.95	21.05	75.00	25.00
**North Carolina (NC)**	76.83	23.17	77.78	22.22
**South Carolina (SC)**	71.43	28.57	≤ 20	≤ 20
**Tennessee (TN)**	57.29	42.71	30.30	69.70
**Virginia (VA)**	81.25	18.75	50.00	50.00
**West Virginia (WV)**	18.75	81.25	100.00	0.00
